# Supplementary material for: Converging synaptic and network dysfunctions in distinct autoimmune encephalitis
Source: EMBO Rep. 2024 Jan 22;25(3):35. doi: 10.1038/s44319-024-00056-2 (PMC10933378; doi:10.1038/s44319-024-00056-2)
Supplement: Supplementary file 2 — Expanded View Figures [file 44319_2024_56_MOESM2_ESM.pdf]

Expanded View Figure

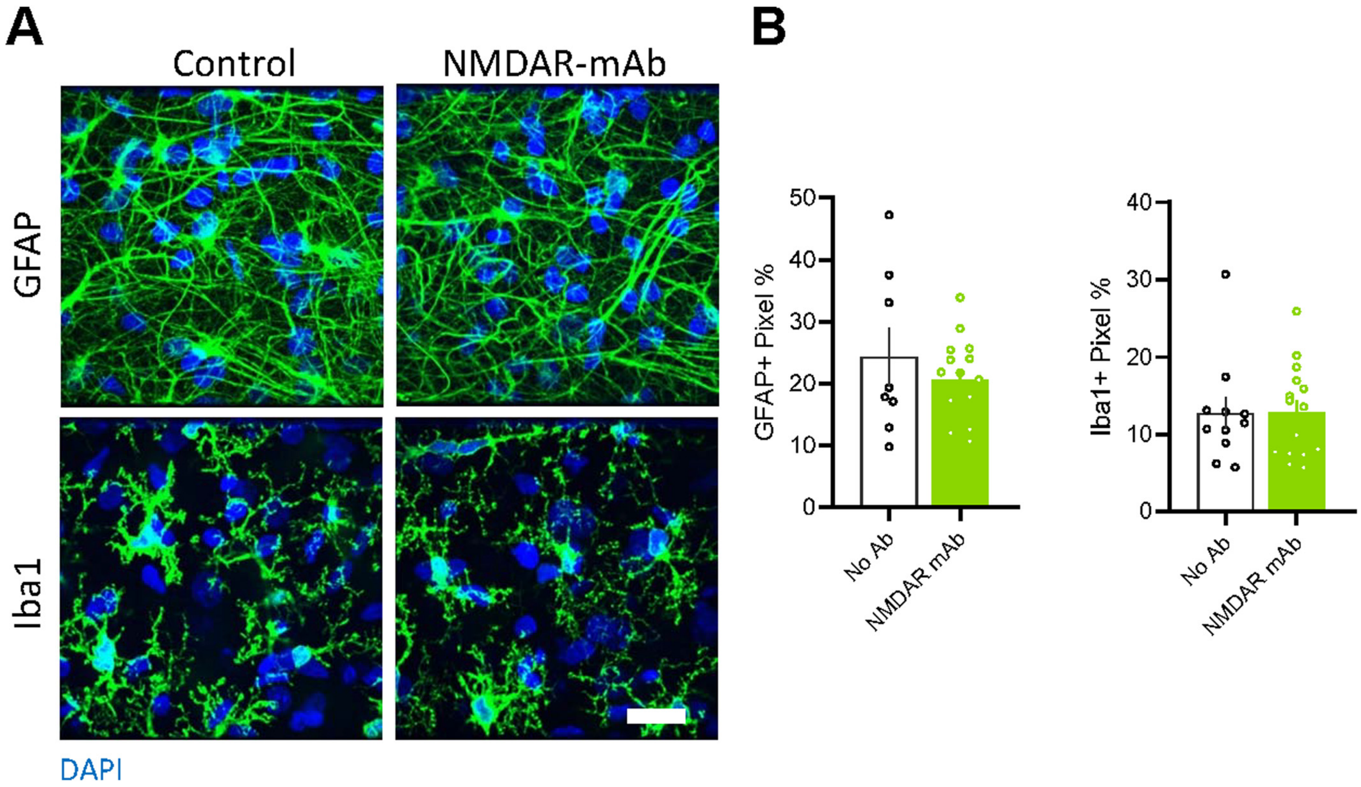

Figure EV1. NMDAR mAb do not alter glial and microglial coverage.

(**A**) Immunocytochemical staining of GFAP-positive and Iba1-positive cells (green), with a DAPI counterstaining (blue), in hippocampal slices exposed to buffer (control) or NMDAR mAb. Scale bar = 20 µm. (**B**) Quantification and comparison of the GFAP and Iba1 fluorescence (fraction of positive pixel) between control and NMDAR mAb conditions (No Ab, $n = 9$; NMDAR mAb, $n = 15$; $P > 0.05$ for all stainings). Data information: All error bars represent the standard error of the mean.

© The Author(s)

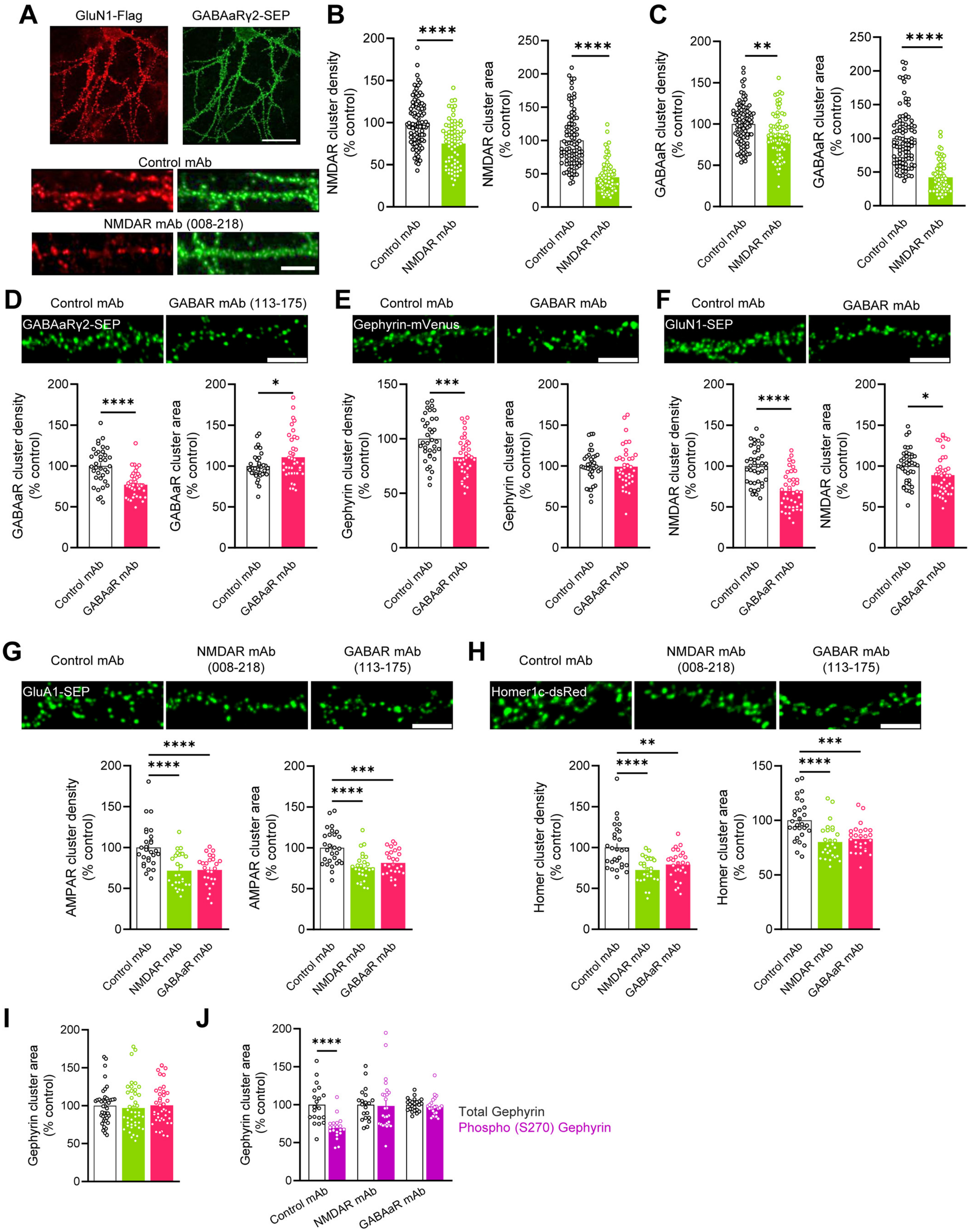

A
GluN1-Flag
GABAaRγ2-SEP
Control mAb
NMDAR mAb (008-218)
B
NMDAR cluster density (% control)
NMDAR cluster area (% control)
C
GABAaR cluster density (% control)
GABAaR cluster area (% control)
D
Control mAb
GABAR mAb (113-175)
GABAaRγ2-SEP
GABAaR cluster density (% control)
GABAaR cluster area (% control)
E
Control mAb
GABAR mAb
Gephyrin-mVenus
Gephyrin cluster density (% control)
Gephyrin cluster area (% control)
F
Control mAb
GABAR mAb
GluN1-SEP
NMDAR cluster density (% control)
NMDAR cluster area (% control)
G
Control mAb
NMDAR mAb (008-218)
GABAR mAb (113-175)
GluA1-SEP
AMPAR cluster density (% control)
AMPAR cluster area (% control)
H
Control mAb
NMDAR mAb (008-218)
GABAR mAb (113-175)
Homer1c-dsRed
Homer cluster density (% control)
Homer cluster area (% control)
I
Gephyrin cluster area (% control)
J
Gephyrin cluster area (% control)
Total Gephyrin
Phospho (S270) Gephyrin
Control mAb
NMDAR mAb
GABAaR mAb

 © The Author(s)

Figure EV2. Monoclonal antibody impacts of macro-organisational cluster area.

(**A**) Immunocytochemical staining of surface NMDAR (GluN1-Flag, red) or GABAaR (Gamma2 SEP, green) in neurons exposed to control mAb or NMDAR mAb (clone 008–218). Scale bars = 20 μm (upper panels), 2 μm lower panels. (**B**, **C**) Mean NMDAR (**B**) or GABAaR (**C**) cluster density and area, normalised to the Control mAb condition (Control mAb $n = 90$ cells; NMDAR mAb $n = 76$; Student t test). (**D–F**) Mean synaptic GABAaR (**D**—Control mAb $n = 36$ cells; GABAR mAb $n = 39$ cells), gephyrin (**E**—Control mAb $n = 36$ cells; GABAR mAb $n = 39$ cells), and NMDAR (**F**—Control mAb $n = 40$ cells; GABAR mAb $n = 42$ cells) cluster density and area in neurons exposed to GABAaR (clone 113–175) mAb (Student t test). Scale bars = 2 μm. (**G**, **H**) Mean synaptic AMPAR (**G**) and Homer1c (**H**) cluster density and area in neurons exposed to Control ($n = 28$ cells), NMDAR (clone 008–218, $n = 27$ cells), or GABAaR (clone 113–175, $n = 27$ cells) mAb (one-way ANOVA). Scale bars = 2 μm. (**I**) Mean gephyrin cluster area, normalised to Control mAb condition (Control mAb $n = 40$ cells; NMDAR mAb $n = 43$ cells; GABAaR mAb $n = 41$ cells; One-way ANOVA). (**J**) Total versus phosphorylated gephyrin puncta density, normalised to the level of total gephyrin staining (Control mAb total gephyrin $n = 20$ cells; Control mAb Phospho-Gephyrin $n = 20$ cells; NMDAR mAb total gephyrin $N = 20$ cells; NMDAR mAb Phospho-Gephyrin $n = 26$ cells; GABAaR mAb total gephyrin $n = 24$ cells; GABAaR mAb Phospho-Gephyrin $n = 24$ cells; Multiple t tests with Benjamini and Hochberg correction for false discovery rate). Data information: All error bars represent the standard error of the mean. Significance levels are represented as *$P < 0.05$, **$P < 0.01$, ***$P < 0.001$ and ****$P < 0.0001$.

© The Author(s)

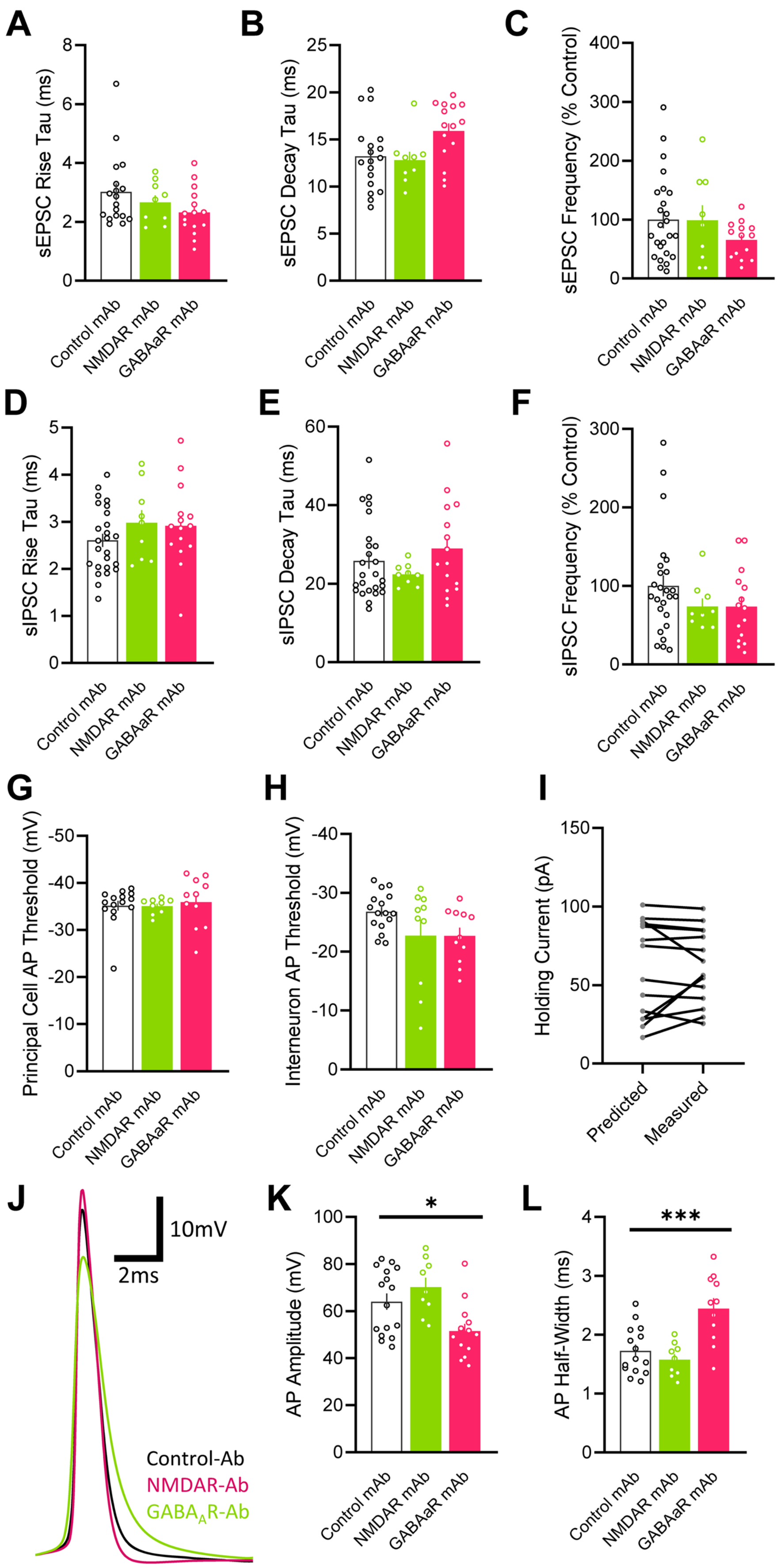

A
sEPSC Rise Tau (ms)
Control mAb
NMDAR mAb
GABAaR mAb
B
sEPSC Decay Tau (ms)
C
sEPSC Frequency (% Control)
D
sIPSC Rise Tau (ms)
E
sIPSC Decay Tau (ms)
F
sIPSC Frequency (% Control)
G
Principal Cell AP Threshold (mV)
H
Interneuron AP Threshold (mV)
I
Holding Current (pA)
Predicted
Measured
J
10mV
2ms
Control-Ab
NMDAR-Ab
GABA$_A$R-Ab
K
AP Amplitude (mV)
*
L
AP Half-Width (ms)
***

 © The Author(s)

Figure EV3. Autoantibodies do not alter synaptic current kinetics or action potential thresholds.

(**A–C**) Mean sEPSC rise and decay tau, and frequency after exposure to control, NMDAR or GABAaR mAb for 24 h (Control mAb $n = 18$ cells; NMDAR mAb $n = 9$ cells; GABAaR mAb $n = 15$ cells; one-way ANOVA). (**D–F**) Mean sIPSC rise and decay tau and frequency after exposure to control, NMDAR or GABAaR mAb for 24 h (Control mAb $n = 25$ cells; NMDAR mAb $n = 9$ cells; GABAaR mAb $n = 15$ cells; one-way ANOVA). (**G**, **H**) Action potential threshold in CA1 hippocampal principal cells (Control mAb $n = 15$ cells; NMDAR mAb $n = 9$ cells; GABAaR mAb n = 11 cells; one-way ANOVA) and interneurons (Control mAb $n = 16$ cells; NMDAR mAb $n = 10$ cells; GABAaR mAb $n = 11$ cells; one-way ANOVA). (**I**) Predicted holding current of mIPSC baseline traces versus experimentally determined holding current ($n = 14$, Student's paired t test). (**J**) Representative action potential traces, collected from CA1 pyramidal cells in current-clamp. (**K**, **L**) Mean action potential amplitude and half-width (Control mAb $n = 16$ cells; NMDAR mAb $n = 19$ cells; GABAaR mAb $n = 14$ cells; one-way ANOVA). Data information: All error bars represent the standard error of the mean. Significance levels are represented as $^*P < 0.05$, $^{**}P < 0.01$, $^{***}P < 0.001$ and $^{****}P < 0.0001$.

© The Author(s)

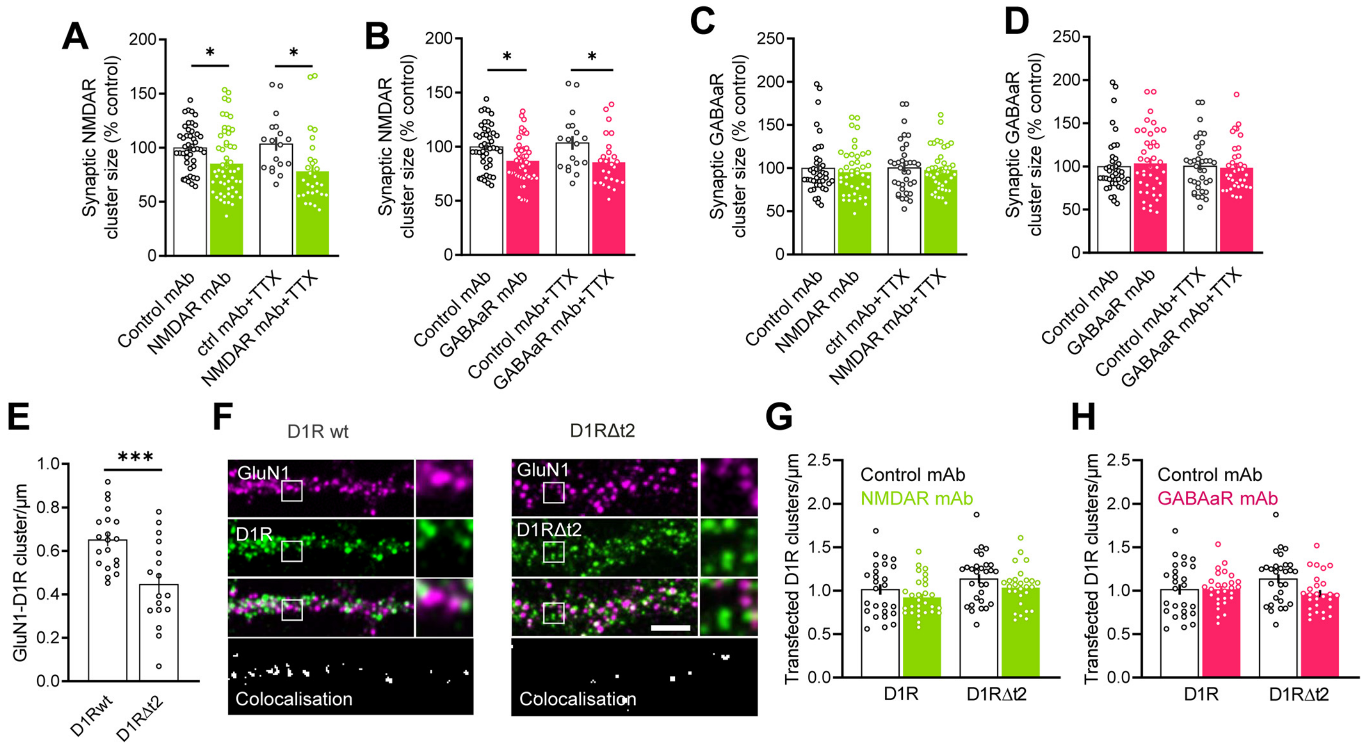

Figure EV4. Excitatory and inhibitory synaptic crosstalk is activity-dependent and D1RΔt2 transfection reduced GluN1-D1R interaction without altering D1R organisation.

(**A**) Cell quantification of synaptic NMDAR cluster density after 24 h exposure to Control mAb or NMDAR mAb, in the presence or absence of TTX (Control mAb $n = 46$ cells; NMDAR mAb $n = 55$; Control mAb +TTX $n = 18$; NMDAR mAb +TTX $n = 29$; one-way ANOVA). (**B**) Cell quantification of synaptic NMDAR cluster density after 24 h exposure to Control mAb or GABAaR mAb, in the presence or absence of TTX (Control mAb $n = 46$ cells; GABAaR mAb $n = 46$; Control mAb +TTX $n = 18$; GABAaR mAb +TTX $N = 26$; one-way ANOVA). (**C**) Cell quantification of synaptic GABAaR cluster density after 24 h exposure to Control mAb or NMDAR mAb, in the presence or absence of TTX (Control mAb $n = 40$ cells; NMDAR mAb $n = 43$; Control mAb +TTX $n = 35$; NMDAR mAb +TTX $n = 43$; one-way ANOVA). (**D**) Cell quantification of synaptic GABAaR cluster density after 24 h exposure to Control mAb or GABAaR mAb, in the presence or absence of TTX (Control mAb $n = 40$ cells; GABAaR mAb $n = 41$; Control mAb +TTX $n = 35$; GABAaR mAb +TTX $n = 37$; one-way ANOVA). (**E**) Mean thresholded GluN1-D1R colocalised cluster density after D1R-WT and D1R-Δt2 transfection (D1R-WT $n = 20$ cells; D1R-Δt2 $n = 17$ cells, Students *t* test). (**F**) Representative immunostainings of D1R-WT and D1R-Δt2 transfected neuronal dendrites expressing GluN1 and D1Rs. Scale bar = 10 µm. (**G**) Mean D1R cluster density after transfection with D1R-WT and D1R-Δt2 constructs and exposure to Control or NMDAR mAb (D1R: Control mAb $n = 26$ cells; NMDAR mAb $n = 29$ cells; D1R-Δt2: Control mAb $n = 29$ cells; NMDAR mAb $n = 28$ cells; one-way ANOVA). (**H**) Mean D1R cluster density after transfection with D1R-WT and D1R-Δt2 constructs and exposure to Control or GABAaR mAb (D1R: Control mAb $n = 26$ cells; GABAaR mAb $n = 28$ cells; D1R-Δt2: Control mAb $n = 29$ cells; GABAaR mAb $n = 27$ cells; one-way ANOVA). Data information: All error bars represent the standard error of the mean. Significance levels are represented as *$P < 0.05$, **$P < 0.01$, ***$P < 0.001$ and ****$P < 0.0001$.

 © The Author(s)

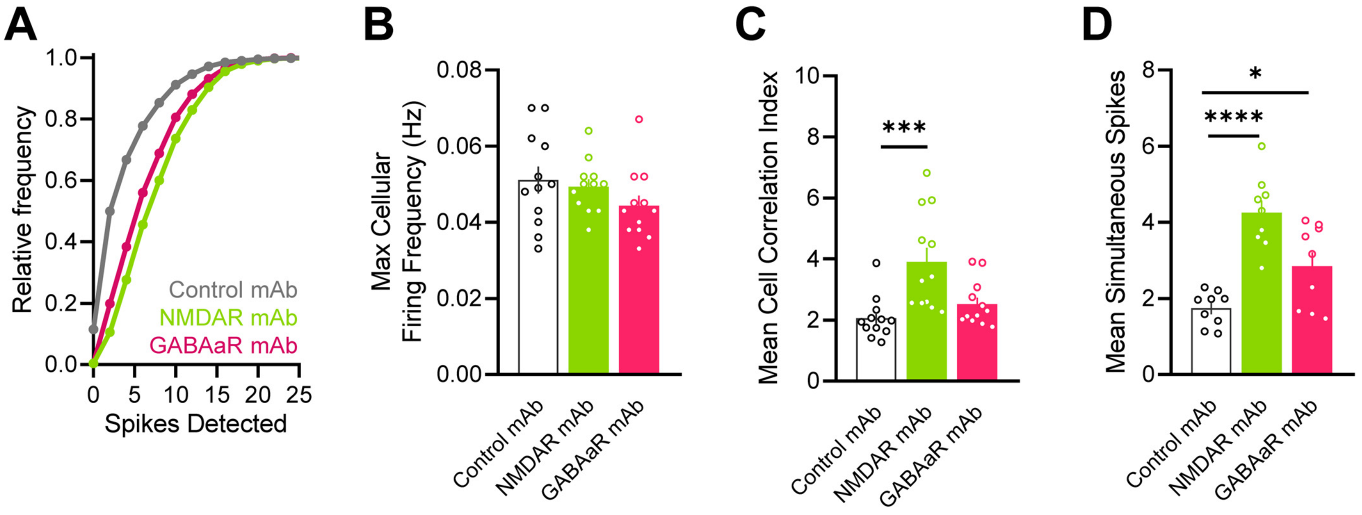

Figure EV5. Autoantibody exposure impacts cell firing and network synchrony.

(**A**) Cumulative frequency distributions of cellular somatic calcium transients from CA1 principal cells exposed to Control, NMDAR and GABAaR mAb (Control mAb $n = 1946$ spikes; NMDAR mAb $n = 2177$ spikes; GABAaR mAb $n = 2163$ spikes). (**B**) Maximum cellular firing frequencies from CA1 principal cells in recorded networks (Control mAb $N = 12$ slices; NMDAR mAb $n = 12$ slices; GABAaR mAb $n = 12$ slices; one-way ANOVA). (**C**, **D**) Mean cell correlation index and simultaneous spike rate for all CA1 principal cells across a recorded network (Control mAb $n = 12$ slices; NMDAR mAb $n = 12$ slices; GABAaR mAb $n = 12$ slices; one-way ANOVA). Data information: All error bars represent the standard error of the mean. Significance levels are represented as *$P < 0.05$, **$P < 0.01$, ***$P < 0.001$ and ****$P < 0.0001$.

© The Author(s)
